# A snapshot of the ongoing clinical research on COVID-19

**DOI:** 10.12688/f1000research.23843.1

**Published:** 2020-05-18

**Authors:** Daniele Piovani, Claudia Pansieri, Laurent Peyrin-Biroulet, Silvio Danese, Stefanos Bonovas

**Affiliations:** 1 Department of Biomedical Sciences, Humanitas University, Pieve Emanuele, Milano, 20090, Italy; 2 Humanitas Clinical and Research Center - IRCCS, Rozzano, Milan, 20089, Italy; 3 Nancy University Hospital, University of Lorraine, Vandoeuvre-lès-Nancy, France

**Keywords:** SARS-CoV-2, 2019-nCoV, 2019 novel coronavirus, severe acute respiratory syndrome coronavirus 2, Covid-19

## Abstract

The pandemic of coronavirus disease 2019 (COVID-19) presents an unprecedented challenge to rapidly develop new diagnostic, preventive and therapeutic strategies. Currently, thousands of new COVID-19 patients are quickly enrolled in clinical studies. We aimed to investigate the characteristics of the COVID-19 studies registered in ClinicalTrials.gov and report the extent to which they have incorporated features that are desirable for generating high-quality evidence.

On April 28, 2020, a total of 945 studies on COVID-19 have been registered in ClinicalTrials.gov; 586 studies are interventional (62.0%), the most frequent allocation scheme is the parallel group assignment (437; 74.6%), they are open-label and the most common primary purpose is the research on treatment.

Too many of the ongoing interventional studies have a small expected sample size and may not generate credible evidence at completion. This might lead to a delayed recognition of effective therapies that are urgently needed, and a waste of time and resources. In the COVID-19 pandemic era, it is crucial that the adoption of new diagnostic, preventive and therapeutic strategies is based upon evidence coming from well-designed, adequately powered and carefully conducted clinical trials.

## Introduction

The pandemic of coronavirus disease 2019 (COVID-19) caused by the novel severe acute respiratory syndrome coronavirus 2 (SARS-CoV-2) presents an unprecedented challenge to rapidly develop new diagnostic, preventive and therapeutic strategies ^1^. Currently, thousands of new COVID-19 patients present for care every day, and many are quickly enrolled in clinical studies. We aimed to investigate the characteristics of the COVID-19 studies registered in ClinicalTrials.gov ^2^, and report the extent to which they have incorporated features that are desirable for generating high-quality evidence.

## Methods

We investigated the ClinicalTrials.gov website on April 28, 2020, using the search term: SARS-CoV-2 OR 2019-nCoV OR 2019 novel coronavirus OR severe acute respiratory syndrome coronavirus 2 OR Covid-19. No restrictions were applied. No screening of trials was performed; all results were included regardless of their content.

Stata 15.0 (Stata Corp., College Station, TX, USA) was used for the analysis of study characteristics.

## Results

A total of 945 studies on COVID-19 have been registered in ClinicalTrials.gov up to April 29, 2020; 586 studies are interventional (62.0%), and 435 of them (74.2%) are randomized. Among interventional studies, the most frequent allocation scheme is the parallel group assignment (437; 74.6%), followed by single group (111; 18.9%], sequential (18; 3.1%), factorial (9; 1.5%), and cross-over assignment (11; 1.9%). The majority of the clinical trials are open-label (no masking, 338 [57.7%]); however, 57 (9.7%) trials are double-blinded, 41 (7.0%) triple-blinded, 90 (15.4%) quadruple-blinded, and 60 (10.2%) single-blinded. Among observational studies, cohort (222; 64.3%) is the most common study design ( Table 1).

**Table 1.** Characteristics of COVID-19 studies registered in ClinicalTrials.gov (n=945).

Study type	N (%)
Study design (n=945)	
Interventional	586 (62.0)
Observational	345 (36.5)
Expanded access	14 (1.5)
Recruitment status (n=945)	
Recruiting or enrolling by invitation	453 (47.9)
Not yet recruiting	414 (43.8)
Active, not recruiting	24 (2.5)
Completed	27 (2.9)
Withdrawn, terminated or suspended	13 (1.4)
Available	13 (1.4)
Intervention type (n=945)	
Drug	405 (42.9)
Biological (cells, blood sampling, etc)	74 (7.8)
Diagnostic test	60 (6.3)
Device	44 (4.7)
Procedure	18 (1.9)
Behavioral	20 (2.1)
Dietary supplement	9 (1.0)
Other/Unknown	315 (33.3)
Target age (n=945)	
Any age	165 (17.5)
Child (<18 y)	5 (0.5)
Child and adult (<65 y)	8 (0.8)
Adult (18–65 y)	35 (3.7)
Adult and elderly (≥18 y)	720 (76.2)
Elderly (≥66 y)	12 (1.3)
Funding (n=945)	
NIH or federal	13 (1.4)
Industry	82 (8.7)
Industry plus other	63 (6.7)
Other (organizations, universities, individuals)	787 (83.2)
Expected trial size (n=931)	200 (66–504)
0–100	344 (37.0)
101–1000	439 (47.1)
>1000	148 (15.9)
Interventional (n=586) [median (IQR)]	150 (52–420)
Observational (n=345) [median (IQR)]	300 (100–1,000)
Study results (n=945)	
Not available	945 (100)
**Interventional studies (n=586)**	
Study phase (n=586)	
Phase 0, 1, 1/2	62 (10.6)
Phase 2, 2/3	212 (36.2)
Phase 3, 4	165 (28.1)
Not applicable	147 (25.1)
Model (n=586)	
Parallel assignment	437 (74.6)
Single group assignment	111 (18.9)
Sequential	18 (3.1)
Factorial assignment	9 (1.5)
Crossover assignment	11 (1.9)
Masking (n=586)	
Open label or no masking	338 (57.7)
Single-blind	60 (10.2)
Double-blind	57 (9.7)
Triple-blind	41 (7.0)
Quadruple-blind	90 (15.4)
Study allocation (n=586)	
Randomized	435 (74.2)
Non-randomized	53 (9.1)
Unknown/missing	98 (16.7)
**Observational studies (n=345)**	
Observational model (n=345)	
Cohort	222 (64.3)
Case-control	34 (9.9)
Case-only	45 (13.0)
Ecologic or community	11 (3.2)
Other	33 (9.6)
Time perspective (n=345)	
Prospective	230 (66.7)
Retrospective	58 (16.8)
Cross-sectional	31 (9.0)
Other	26 (7.5)

Most studies target adult or elderly participants, while 178 (18.8%) enroll children, with only five (0.5%) recruiting exclusively children. Median expected study size is 200 (interquartile range, 66–504), although sample sizes vary from ≤100 (344; 37.0%) to >1,000 individuals (148; 15.9%). Overall, only 27 of 945 studies (2.9%) have completed recruitment, 453 (47.9%) are actively recruiting subjects, while a large number of studies (414; 43.8%) are not yet actively recruiting participants. Most of the studies are conducted in Europe (n=327), North America (n=217, of which 186 in the US), East Asia (n=102), Africa (n=27), and in South America (n=26). No study has reported results yet.

Among the interventional studies, the most common primary purpose is the research on treatment (441; 75.3%), followed by prevention (79; 13.5%), supportive care studies (22; 3.8%), and diagnostic investigations (17; 2.9%). Regarding the drugs under scrutiny, hydroxychloroquine (110; 28.6%), azithromycin (38; 9.9%), lopinavir/ritonavir (24; 6.2%), interferon-α and -β (24; 6.2%), glucocorticoids (22; 5.7%), chloroquine (14; 3.6%), favipiravir (10; 2.6%), remdesivir (8; 2.1%), tocilizumab (21; 5.5%), anti-SARS-CoV-2 immunoglobulins (15; 3.9%) and sarilumab (9; 2.3%) account for the majority of interventional studies. Additional details are featured in Table 2.

**Table 2.** Characteristics of COVID-19 interventional studies registered in ClinicalTrials.gov (n=586).

Study type	No. (%)
Primary purpose (n=586)	
Treatment	441 (75.3)
Prevention	79 (13.5)
Supportive care	22 (3.8)
Diagnostic	17 (2.9)
Other	13 (2.2)
Screening	5 (0.8)
Basic science	5 (0.8)
Health services research	4 (0.7)
Drugs (n=385)	
* Repurposed drugs*	
Hydroxychloroquine	110 (28.6)
Azithromycin	38 (9.9)
Lopinavir/Ritonavir	24 (6.2)
Glucocorticoids	22 (5.7)
Interferon-α and -β	24 (6.2)
Chloroquine	14 (3.6)
Nitazoxanide	8 (2.1)
Camostat	4 (1.0)
Oseltamivir	4 (1.0)
Ribavirin	1 (0.3)
*Investigational agents*	
Favipiravir	10 (2.6)
Remdesivir	8 (2.1)
*Adjunctive therapies*	
Tocilizumab	21 (5.5)
Anti SARS-CoV-2 immunoglobulins	15 (3.9)
Sarilumab	9 (2.3)

## Discussion

Our survey presents the current COVID-19 clinical research landscape. Several hundreds of clinical studies have been initiated all over the globe, and the number is growing. Most interventional studies incorporate randomisation, which is considered the hallmark of high-quality clinical trials ^3^, while more than 40% are blinded.

Studies are being conducted especially in the most affected areas: Europe and US. The number of COVID-19 cases in low to middle-income countries is still relatively low, also reflecting scarce testing, but is expected to rise in the next period. These countries will need more research on organizational measures, and trials on interventions that are affordable and applicable to those settings ^4^.

Most studies focus on adults and elderlies, while only few target children, possibly reflecting the observed burden of the disease. Additional effort is needed to ensure that minors are included in COVID-19 clinical research, so that therapeutic decisions are based upon high-quality evidence.

No drug with proven clinical efficacy currently exists for SARS-CoV-2 infection. Despite the absence of solid evidence, several treatments are being currently used in clinical practice in several countries, with sometimes disastrous consequences ^5^. Too many of the ongoing interventional studies have a small expected sample size, and may not generate credible evidence at completion ^4^. This might lead to a delayed recognition of effective therapies that are urgently needed, and a waste of time and resources. In the COVID-19 pandemic era, it is crucial that the adoption of new diagnostic, preventive and therapeutic strategies is based upon evidence coming from well-designed, adequately powered and carefully conducted clinical trials.

## Data availability

The Clinical Trials website can be accessed here: https://clinicaltrials.gov/
