# Supplementary material for: A snapshot of the ongoing clinical research on COVID-19
Source: F1000Res. 2020 May 18;9:373. [Version 1] doi: 10.12688/f1000research.23843.1 (PMC7256468; doi:10.12688/f1000research.23843.1)
Supplement: Peer review history

# Peer review: Reviewer response for version 1

**DOI:** 10.5256/f1000research.26310.r63547

**Published:** 2020-05-28

**Authors:** Ioannis Mamais

**Affiliations:** 1 Department of Hygiene, Epidemiology and Medical Statistics, Medical School, National and Kapodistrian University of Athens, Athens, Greece

I have read it through, and it seems to be an interesting brief report.

 Some comments below: 

- 74.2% of those studies are mention as "Randomized" and 40% as blinded, from researchers. Authors of this brief report can "discuss" those numbers and prepare readers that most of those may have a high risk of bias (maybe with no control group, or bias arising from the randomisation process, etc).
- "Additional effort is needed to ensure that minors are included in COVID-19 clinical research, so that therapeutic decisions are based upon high-quality evidence". I am not very sure if it is necessary to discuss this in the brief report

Is the work clearly and accurately presented and does it cite the current literature?

Yes

If applicable, is the statistical analysis and its interpretation appropriate?

Not applicable

Are all the source data underlying the results available to ensure full reproducibility?

Yes

Is the study design appropriate and is the work technically sound?

Yes

Are the conclusions drawn adequately supported by the results?

Yes

Are sufficient details of methods and analysis provided to allow replication by others?

Partly

Reviewer Expertise:

Molecular Epidemiology

I confirm that I have read this submission and believe that I have an appropriate level of expertise to confirm that it is of an acceptable scientific standard.

# Peer review: Reviewer response for version 1

**DOI:** 10.5256/f1000research.26310.r63548

**Published:** 2020-05-26

**Authors:** Demetris Lamniosos

**Affiliations:** 1 Department of Health Sciences, School of Sciences, European University Cyprus, Nicosia, Cyprus

This brief report is a survey of the current COVID-19 clinical research landscape. The number of clinical studies on COVID-19 is rapidly growing and it is important the investigation of whether these studies are incorporating features that are desirable for generating high-quality evidence.

 This survey performed this investigation and found that too many of the ongoing interventional studies have a small expected sample size. This might lead to delayed recognition of effective therapies and a waste of time and resources. This important evidence should guide the design of any future clinical study on COVID-19 and the decision of any funding body as well as the approval of any bioethics committee. For this reason, I consider this brief report important for the scientific community.

 There are a few minor suggestions for the authors:

- Table 1: The presentation of the studies' characteristics will be improved if they are presented separately for interventional and observational studies (particularly for the intervention type and expected study size). In this way, the table will provide more information about the studies' characteristics for each study type (interventional and observational).
- Table 1: It will be useful to report the type of randomization (such as blocking and block size).

Is the work clearly and accurately presented and does it cite the current literature?

Yes

If applicable, is the statistical analysis and its interpretation appropriate?

Not applicable

Are all the source data underlying the results available to ensure full reproducibility?

Yes

Is the study design appropriate and is the work technically sound?

Yes

Are the conclusions drawn adequately supported by the results?

Yes

Are sufficient details of methods and analysis provided to allow replication by others?

Yes

Reviewer Expertise:

Epidemiology, Statistics, Research Methods, Public Health

I confirm that I have read this submission and believe that I have an appropriate level of expertise to confirm that it is of an acceptable scientific standard.
